# Heart and Head: Profiles and Predictors of Self-Assessed Cognitive and Affective Empathy in a Sample of Medical and Health Professional Students

**DOI:** 10.3389/fpsyg.2021.632996

**Published:** 2021-06-16

**Authors:** Laura Giusti, Silvia Mammarella, Anna Salza, Donatella Ussorio, Denise Bianco, Massimo Casacchia, Rita Roncone

**Affiliations:** ^1^Department of Life, Health and Environmental Sciences, University of L'Aquila, L'Aquila, Italy; ^2^Hospital S. Salvatore, University Unit Rehabilitation Treatment, Early Interventions in Mental Health, L'Aquila, Italy

**Keywords:** empathy, medical students, health professional students, depression, loneliness, hope, psychosocial conditions, predictors and associations

## Abstract

For medical and health professions, students learning to respond to others' distress with well-regulated empathy is an important developmental skill linked to positive health outcomes and professionalism. Our study aimed to investigate the sociodemographic, psychological, and psychosocial differences between medical (MS) and health professional (HPS) students and their empathic abilities, since both populations share common stressors, namely, dealing with suffering people. Additionally, we were interested in assessing the psychological and psychosocial predictors of empathy of MS compared to HPS. One hundred thirty MS and 86 HPS were administered the Patient Health Questionnaire-9, Interpersonal Reactivity Index, Integrative Hope Scale, and UCLA Loneliness Scale. The two groups showed differences in their contextual characteristics, with the HPS group having larger families, lower parents' education levels, and lower family income compared to the MS group. In both groups, ~15% of students reported previous contact for psychological problems. A higher proportion of HPS (23.3%) reported depressive symptoms than MS (10%), and female HPS reported more intense feelings of loneliness than other subgroups of students. No differences were found between the two groups in self-assessed cognitive and affective empathy. In both groups, women showed greater affective scores than men and, at the same time, seemed to be particularly prone to personal distress. The cognitive empathic dimension of “perspective taking” was predicted by young age (OR, 612; 95% CI, 1.395–15.242) and the overall socioeconomic status (OR, 3.175; 95% CI, 1.154–8.734) of the HPS. Self-assessed affective competence was predicted by female gender (OR, 3.112; 95% CI, 1.328–7.288), depressive symptomatology (OR, 2.777; 95% CI, 1.004–7.681), higher mother's level of education (OR, 2.764; 95% CI, 1.147–6.659), and feeling of hope related to social relationships (OR, 1.367; 95% CI, 1.152–1.622). Risk factors for poor self-assessed affective emphatic skills were previous contact for psychological problems (OR, 3.263; 95% CI, 1.238–8.601) and feelings of loneliness (OR, 1.18; 95% CI, 1.09–1.276). Our findings emphasize the need to test psychosocial models to better understand empathic skills.

## Introduction

Learning to respond to others' distress with well-regulated empathy is an important developmental skill linked to positive health outcomes and professional abilities (Tone and Tully, 2014). Findings from studies targeting age groups from infancy through adulthood have suggested that empathic emotions, thoughts, and behaviors emerge and evolve in most individuals based on fairly predictable developmental patterns (i.e., gender and age differences) (Tone and Tully, 2014). In its typical or adaptive form, affective empathy leads to compassionate responses to others' emotional states (Shamay-Tsoory et al., 2009); the cognitive aspect of empathy is generally associated with positive social behaviors, such as cooperation, provision of social support, and volunteering (Verhofstadt et al., 2008).

Empathy is a crucial humanistic component of patient care (Hojat et al., 2011) that provides efficient and patient-centered clinical encounters (Larson and Yao, 2005; Veloski and Hojat, 2006). Positive correlations have been found between physicians' self-reported empathy and patient outcomes (Hojat et al., 2011). Moreover, empathetic doctors are more satisfied with their jobs and less susceptible to burnout and depression (Thirioux et al., 2016).

Patients benefit when all members of the health care team provide empathic care, but despite the relevance of empathy in patient outcomes (Hojat et al., 2011; Del Canale et al., 2012), empirical research on empathy among different health professionals is scarce, and comparisons between health professionals and physicians are rare. The results of the few studies carried out have been contradictory: some authors did not find significant differences in empathic scores between nurses and physicians (Fields et al., 2004), while Williams et al. (2015) found that medical students scored higher than nursing students. A recent review by Charitou et al. (2019) considered 22 studies that measured levels of empathy in a variety of health professionals (e.g., nurses and midwives), medical students and physicians. In most studies, women had higher levels of empathy than men, which was true for both students and health professionals. Although most of the literature is based on the empathic abilities of medical student populations, many of the stressors associated with university life and clinical placements common in medical student training will be present in the training of all health professionals (McConville et al., 2017), who are crucial resources in the organization of health services.

Since empathy is a crucial ability for establishing a positive relationship with service users and ensuring better treatment outcomes, many studies have investigated empathic abilities in medical students and the association of their well-being during their academic careers, highlighting how empathic abilities decrease over time (Bellini and Shea, 2005; Neumann et al., 2011; Piumatti et al., 2020). There are many potential psychological barriers to empathy, such as student depression, burnout, and low quality of life or wellness behaviors (Damiano et al., 2017).

Medical students suffer an increased risk of depression compared to their peers currently enrolled in non-medical university courses (Rotenstein et al., 2016; Singh et al., 2016; Tam et al., 2019; Atienza-Carbonell and Balanza-Martinez, 2020), and the presence of depressive symptoms seems to occur as early as the 1st year of the students' medical education (Grace, 2018), especially in women.

The conditions of medical university students have also been a research focus in Italy (Messina et al., 2016; Pighi et al., 2018; Volpe et al., 2019). Italian surveys have found that medical students highlighted issues associated with anxiety and depression, emotional distress (Volpe et al., 2019), low perceived quality of life (Messina et al., 2016), problems related to alcohol consumption, and the propensity to use substances as cognitive enhancers (Pighi et al., 2018).

Psychological distress (e.g., burnout, anxiety, and depressive symptoms) appears to be one of the most important causes of empathy decline. Some studies have shown that in medical students, lower empathy scores were correlated with symptoms of depression, especially in female students (Thomas et al., 2007; Neumann et al., 2011).

Some authors have reported a negative association between loneliness and empathy (Marilaf Caro et al., 2017; Soler-Gonzalez et al., 2017) and an inverse association between empathy and distress (San-Martín et al., 2016; Marilaf Caro et al., 2017; Yuguero et al., 2017). A recent study found a significant interaction between depression and loneliness in predicting suicide risk in a sample of college students (Chang et al., 2019).

If loneliness represents a barrier to developing empathy, the feeling of hope could be considered a facilitator of empathic skills. Hope represents a powerful predictor of quality of life, and it is considered an essential factor associated with well-being (Slade, 2010). In a sample of medical students, the presence of hope reduced the perception of psychological distress (Krageloh et al., 2015; Heinen et al., 2017).

In the last 30 years, many studies have emphasized the important role that the family environment plays in healthcare professionalism, with special attention given to the development of empathic abilities (Bernabeo et al., 2018; Berduzco-Torres et al., 2020).

It is well-known that a parent's educational level influences the realistic expectations and the ideal educational aspirations of the student and that parental educational attainment has long-term influences on students' educational attainments (Gooding, 2001). Mothers' educational attainment levels did not directly affect students' academic achievement as much as fathers' educational attainment levels, but indirectly, they impacted the psychosocial maturity and level of independence of students, which in turn determined levels of achievement.

A positive parental pattern seems to significantly contribute to the development and enhancement of empathetic abilities of nursing students engaged in patient care (Li et al., 2018). Medical students who were satisfied with their relationship with their mothers scored higher than those who were neutral or not satisfied (Hasan et al., 2013).

On this basis, we investigated the sociodemographic, psychological, and psychosocial differences and the level of empathy of medical students (MS) compared to health professional students (HPS) since both of these populations share common stressors, namely, dealing with suffering people. We were also interested in assessing the psychosocial (family size, parents' education, family financial condition, working student status) and psychological (pre-existing psychological problems, depression, feeling of loneliness, and hope) predictors of cognitive and affective empathic abilities in these two populations.

Additionally, with consideration of a psychosocial vulnerability model that emphasizes the role of individual difference variables and contextual variables (O'Neil and Emery, 2002), we proposed two hypotheses: (1) HPS and MS would exhibit the same levels of depression and empathy; (2) the levels of cognitive and affective empathy can be independently predicted from age, gender and socioeconomic and cultural level in these samples.

## Materials and Methods

### Sample

The study was conducted at the *Counselling and Consultation Services for Students (*S.A.C.S.) at the University of L'Aquila, Italy. The mission of the S.A.C.S. is to monitor the student's well-being status, to identify students' personal and academic difficulties and to help them by offering psychological support.

Students were assessed by the multidisciplinary S.A.C.S. team consisting of psychiatrists, residents in psychiatry, psychologists, cognitive-behavioral psychotherapists, and psychiatric rehabilitation technicians. The survey included a purposive sample, i.e., students attending the last years of the courses, which is the 5th year for medical students (MS group) and the second and third years for health professional students (HPS group). The first group was selected because of their advanced traineeship experience with patients; the second group was selected because, having their traineeship in the rehabilitation area, they were involved with patients in medium- and long-term treatments. The second-level six-year degree course in Medicine and Surgery provides for the acquisition of 60 internship European Credit System, ECTS, credits from the 3rd to the 6th year of the program course. In the first-level health professional degree courses, the traineeship accounts for 60 ECTS credits out of the 3-year 180 ECTS credits of the program course.

The current study was approved by the Internal Review Board Committee of the University of L'Aquila. The participants provided their written informed consent to participate in this study.

The questionnaires were administered in paper and pencil form during the index week January 13–18, 2020, at the end of the first semester of the A.Y. 2019–20. The member of the S.A.C.S. team oversaw the administration of tests that were filled anonymously by the attending students. The administration of the questionnaires was conducted in the classroom at the end of the lessons.

All these courses have a limited number of available seats established annually by the Ministry of University and Scientific and Technological Research. At the University of L'Aquila, the medical second-level degree course enrolls ~140 students per year; physiotherapy degree course, 45 students; neuropsychomotor therapy in developmental age degree course, 30 students; and psychiatric rehabilitation technique degree course, 25 students.

### Instruments

All participants completed a form for sociodemographic and clinical data collection and were administered the following battery of psychological measures.

The **Interpersonal Reactivity Index (IRI)** (Davis, 1983; Albiero et al., 2006) is a 28-item self-report instrument rated on a five-point Likert scale (from 1 = never true to 5 = always true) investigating cognitive and affective components of the construct of empathy. The cognitive dimension includes the “perspective taking” subscale (PT), which assesses the tendency to spontaneously adopt the psychological viewpoint of others, and the “fantasy scale” subscale (FS), which assesses tendencies to transpose themselves imaginatively into the feelings and actions of fictitious characters in books, movies, and plays. On the other hand, the affective dimension includes the “empathetic concern” subscale (EC), which assesses “other-oriented” feelings and concerns for unfortunate others, and the “personal distress” subscale (PD), which measures “self-oriented” feelings of discomfort and negative activation in interpersonal situations of emergency and difficulty. Each dimension includes seven items with possible scores ranging from 7 to 35. Cronbach's alpha values for IRI subscales ranged from 0.70 to 0.83, and correlation coefficients ranged from 0.01 to 0.37 between subscales (Davis, 1980; Siu and Shek, 2005; De Corte et al., 2007; Fernandez et al., 2007). Internal consistency for the IRI was high in this sample for the whole measure (Cronbach's α = 0.80) and within the two affective dimension subscales (empathic concern α = 0.80; personal distress α= 0.78). Lower values were calculated for the two cognitive IRI subscales (perspective taking α = 0.64; fantasy scale α= 0.56). Correlations between subscales of self-assessed empathy by Interpersonal Reactivity Index (IRI) scores are presented in Table 1.

**Table 1:** Correlations between subscales of self-assessed empathy by Interpersonal Reactivity Index (IRI) scores.

		**PT - Perspective-Taking Scale**	**FS –Fantasy Scale**	**EC - Empathic Concern Scale**	**PD - Personal Distress Scale**	**IRI-Interpersonal Reactivity Index, total score**
FS-Fantasy Scale	Pearson's Correlation	0.440^**^	1			
	2-tailed *p*-value	0.000				
EC-Empathic Concern Scale	Pearson's Correlation	0.542^**^	0.505^**^	1		
	2-tailed *p*-value	0.000	0.000			
PD-Personal Distress Scale	Pearson's Correlation	0.121	0.427^**^	0.388^**^	1	
	2-tailed *p*-value	0.077	0.000	0.000		
IRI-Interpersonal Reactivity Index, total score	Pearson's Correlation	0.453^**^	0.648^**^	0.719^**^	0.573^**^	1
	2-tailed *p*-value	0.000	0.000	0.000	0.000	

** *p = 0.001*.

The **Patient Health Questionnaire (PHQ-9)** (Spitzer et al., 1999) contains nine items that are rated on a four-point Likert scale (from 0 = not at all to 3 = nearly every day). The PHQ-9 total score for the nine items can range from 0 to 27. The PHQ-9 is a questionnaire used for the evaluation of depressive symptoms and their severity levels. A PHQ-9 score ≥10 had a sensitivity of 88% and a specificity of 88% for major depression. PHQ-9 scores of 5, 10, 15, and 20 represent mild, moderate, moderately severe, and severe depression, respectively (Kroenke et al., 2001). In this study, we used a cuto? score of 10. The internal reliability was excellent, with a Cronbach's alpha of 0.89 (Kroenke et al., 2001). Internal consistency for the PHQ-9 in our sample was high (Cronbach's α = 0.87).

The **Integrative Hope Scale (IHS)** (Schrank et al., 2011) consists of 23 items rated on a six-point Likert scale (from 1= strongly disagree to 6 = strongly agree). It provides an overall score and four-dimensional scores, obtained by summing up the individual item scores, after reverse coding the negative items. This produces possible overall hope scores ranging from 23 to 138, with higher scores representing higher hopefulness. The scores for the subdimensions vary according to the number of items. The scale's factor structure was highly stable, and its internal consistency was high (alpha = 0.92 for the overall scale and 0.80–0.85 for its four subscales (“trust and confidence”; “lack of perspective”; “positive future orientation”; and “social relations and personal value”) (Schrank et al., 2011). Hope scores were negatively correlated with depression (*r* = 0.68) and positively correlated with quality of life (*r* = 0.57), with the factor analysis and item discriminant validity supporting the scale's construct validity. In our sample the internal consistency of the scale was high (alpha = 0.87 for the overall scale and 0.75–0.87 for its four subscales (“trust and confidence”; “lack of perspective”; “positive future orientation”; and “social relations and personal value”).

The **UCLA Loneliness Scale** (Russell, 1996) is the most widely used instrument to assess loneliness. This scale consists of 11 items worded in a negative/lonely direction and 9 items worded in a positive/non-lonely direction. The participants rated the extent of their agreement with these questions on a 4-point Likert scale ranging from 1 (never) to 4 (often). The participants' scores were calculated by reverse coding the 9 positive items and then summing all 20 items; higher scores indicated greater loneliness. The measure is highly reliable, both in terms of internal consistency (coefficient alpha ranging from 0.89 to 0.94) and test-retest reliability over a 1-year period (*r* = 0.73) (Russell et al., 1980).

In this sample internal consistency for the UCLA Loneliness Scale was high (Cronbach's α = 0.83).

### Statistical Procedures

Descriptive analyses were performed for all investigated variables.

ANOVA and chi-square analyses were conducted to investigate sociodemographic and psychological differences between the MS and HPS groups and the four groups based on gender stratification. Bonferroni *post-hoc* tests were performed to identify statistically significant differences among the four groups.

Logistic regression analyses were conducted to identify potential predictors of empathy using a predictive psychosocial model. Each of the four components of empathy (2 cognitive, i.e., “perspective taking” and “fantasy,” and 2 affective, i.e., “empathic concern” and “personal distress”), as assessed by the IRI, was investigated for its predictive value.

We calculated the 75th percentile of the 4 dimension scores to evaluate the higher expression of empathy for the 2 cognitive dimensions and affective dimension of “empathic concern” and for the barrier of “personal distress.”

First, sociodemographic and family condition data (gender, age group, size of the family, parents' education, and family financial condition) were included as potential predictors. Age was coded into 2 categories (18–21 years and 22 years and above). The age range 18–21 years was selected as an entry in the “majority age” since 21 represented a reference term in Italy. Until 1975, it represented the age for the right to vote, which was subsequently reduced to 18 years. This categorization was based on the assumption that younger students may be more prone to show empathy than older students. Family size was coded into 2 categories (2–4 members and 5 or more members) since we hypothesized that being part of a larger family could be a live-and-learning model of affective skills. Based on the Report on Natality and Fecundity of the Italian resident population, the year 2019, with an average of 1.18 children born from women of Italian citizenship (Italian National Statistics Institute, 2019), having 3 children or more (and thus being 5 people in the family) is the current cutoff in the Italian statistical report. Parents' education was coded into two categories (no university degree and university degree). High education of students' parents was suspected to contribute to developing the cognitive dimension of empathy. The students' family financial condition was coded into 2 categories (high-medium and low financial condition, e.g., the family cannot afford holidays and has to limit daily expenses).

Second, two student variables (being a health professional and a working student) were included as potential predictors and coded into 2 categories (no/yes).

Third, we entered variables related to the students' psychological conditions (each was coded into 2 categories: yes/no): previous contact for psychological problems and depressive symptomatology, as assessed by PHQ-9 scores >10. Feelings of loneliness as measured by the UCLA Loneliness Scale and feelings of hope in social relationships and personal value as assessed by a specific IHS subscale were included as continuous variables.

We calculated odds ratios (OR) with 95% confidence intervals for the logistic regression analysis.

Statistical analyses were carried out using SPSS 26.0 for Windows.

## Results

The main sociodemographic characteristics of the sample are reported in Table 2.

**Table 2:** Socio-demographic and clinical characteristics of the sample (*n* = 216).

	**Medical students (*n* = 130)**	**Health professional students (*n* = 86)**	
**Gender (%)**			
- Men	48 (36.9)	32 (37.2)	
- Women	82 (63.1)	54 (62.8)	
			Chi-square: 0.002; d.f. 1; *p =* 0.966
**Age, mean (SD)**	24.7 (1.53)^*^	22.9 (4.0)	
			ANOVA: *F =* 20.802; *p =* 0.000
Family composition (%)^*^			
1–2 members	25 (19.3)	7 (8.1)	
3 members	31 (23.8)	20 (23.3)	
4 members	54 (41.5)	32 (37.2)	
5 members	14 (10.8)	19 (22.1)	
>5 members	6 (4.6)	8 (9.3)	
			Chi-square: 10.648; d.f. 4; *p =* 0.031
Mother's education, years (%)^**^			
**-**5 years education	2 (1.5)	1 (1.2)	
8 years education	12 (9.2)	22 (25.9)	
13 years education	59 (45.4)	45 (52.9)	
17 years education	57 (43.9)	17 (20)	
			Chi-square: 18.158; d.f. 3; *p =* 0.000
Father's education, years (%)^**^			
5 years education	2 (1.5)	2 (2.3)	
8 years education	13 (10)	25 (29.1)	
13 years education	59 (45.4)	39 (45.3)	
17 years education	56 (43.1)	20 (23.3)	
			Chi-square: 16.652; d.f. 3; *p =* 0.001
Family income/Status of income (%)^**^			
Low	8 (6.2)	15 (17.4)	
Medium	42 (32.3)	47 (54.7)	
High	80 (61.5)	24 (27.9)	
			Chi-square: 24.624; d.f. 2; *p =* 0.000
Working students (%)^**^	10 (7.7)	17 (19.8)	
			Chi-square: 6.900; d.f. 1; *p =* 0.009
**Academic career in progress (%)**			
Medicine	130 (100)		
Physiotherapist		45 (52.4)	
Child Neuropsychomotor Therapists in developmental age		23 (26.7)	
Psychiatric Rehabilitation Technicians		18 (20.9)	
Previous contact for psychological problems^**^(%)			
Any contact	111 (85.4)	73 (84.9)	
General practitioners	14 (10.8)	0	
Private professionals-mental health services	5 (3.8)	13(15.1)	
			Chi-square: 17.152; d.f. 2; *p =* 0.000

* *p = < 0.05*;

** *p = < 0.001*.

Two hundred and sixteen students (130 medical students, MS; 86 health professional students, HPS) responded to the investigation promoted by the Counselling and Consultation Services for Students (S.A.C.S.) of the University of L'Aquila. Health professional courses included physiotherapy, neuropsychomotor therapy in the developmental age degree program, and psychiatric rehabilitation techniques. Of the 240 students enrolled in these courses, the majority (216) agreed to participate, and only 10% (*n* = 24) did not complete the questionnaires. In both groups, more than 60% of students were women. A statistically significant difference was found in the age of the “older” students in the MS group. The family composition showed a significantly greater proportion of larger families in the HPS group. In the MS group, the parents' education level showed a higher percentage of graduates and a higher family income compared to the MS group.

Working students were significantly more represented in the HPS group, at approximately one-fifth of the sample.

In both groups, ~15% of students reported previous contact for psychological problems, with some differences in relation to their request for help, namely, a larger proportion of MS interacted with general practitioners and HPS used mental health services and private professionals.

### Interpersonal Reactivity Index (IRI)

Regarding empathetic and interpersonal abilities, no statistically significant differences were found between the two groups in the cognitive and affective dimensions (Table 3). Based on gender, statistically significant differences were found (Table 4). The men belonging to the MS group showed lower scores on the **empathetic concern scale** than the women in the MS group (*post-hoc* Bonferroni test; mean difference = −2.944; *p* < 0.016) and the women in the HPS group (mean difference = −3.484; *p* < 0.007). On the **personal distress scale**, the women in the MS group showed significantly higher scores than their male course colleagues (mean difference = 2.698; *p* < 0.014) and the men in the HPS group (mean difference = 2.667; *p* < 0.048). On the same scale, the women in the HPS group showed significantly higher scores than their male course colleagues (mean difference = 3.094; *p* < 0.026) and the men in the MS group (mean difference = 3.125; *p* < 0.008).

**Table 3:** Means and standard deviations of clinical and psychological measures of total sample (*n* = 216).

	**Medical** ** students** ** (*n =* 130)**	**Health professional students** ** (*n =* 86)**	
**IRI-Interpersonal Reactivity Index m (SD)**			
- PT-Perspective-Taking Scale (7–35)	24.3 (4.7)	24.4 (3.1)	ANOVA: *F =* 0.011; *p =*0.916
- FS-Fantasy Scale (7–35)	21.0 (4.3)	21.1 (4.2)	ANOVA: *F =* 0.012; *p =* 0.911
- EC-Empathic Concern Scale (7–35)	25.1 (5.7)	25.7 (4.1)	ANOVA: *F =* 0.667; *p =* 0.415
- PD-Personal Distress Scale (7–35)	17.0 (4.8)	17.3 (4.4)	ANOVA: *F =* 0.173; *p* = 0.678
**PHQ – Patient Health Questionnaire total score, mean (SD)**	5.72 (4.1)	7.02 (4.6)^*^	ANOVA: *F =* 4.694; *p =* 0.031
**PHQ – Patient Health Questionnaire range (*****n***, %)^*^			
Absence – subthreshold depressive symptoms (1–9)	117 (90)	66 (76.7)	
Mild depressive symptoms (10–14)	9 (6.9)	13 (15.1)	
Moderate depressive symptoms (15–19)	1 (0.8)	5 (5.8)	
Severe depressive symptoms (>20)	3 (2.3)	2 (2.4)	
			Chi-square: 9.227; d.f. 3; *p =* 0.026
**HIS – Hope Integrative Scale total score, mean (SD)**	86.2 (12.3)	85.4 (11.7)	ANOVA: *F =* 0.255; *p =* 0.614
- Trust and Confidence (9–54)	31.7 (5.3)	31.5 (5.6)	ANOVA: *F =* 0.050; *p =* 0.824
- Social Relationship and Personal Value (4–24)	14.7 (2.8)	14.5 (2.3)	ANOVA: *F =* 0.332; *p =* 0.565
- Positive Future Orientation (4–24)	17.9 (2.2)	18.1 (1.8)	ANOVA: *F =* 0.304; *p =* 0.582
- Lack of Perspective (6–36)	8.23 (4.9)	8.80 (5)	ANOVA: *F =* 0.792; *p =* 0.374
**UCLA Loneliness Scale total score, mean (SD)**	12.27 (4.7)	13.73 (5.7)^*^	ANOVA: *F =* 4.110; *p =* 0.044

* *p = < 0.05*.

**Table 4:** Means and standard deviations of clinical and psychological measures of total sample by gender (*n* = 216).

	**Medical** ** students** ** (*****n=*** **130)**		**Health professionals** ** students** ** (*****n=*** **86)**		
	**Men(*n =* 48)**	**Women(*n =* 82)**	**Men(*n =* 32)**	**Women(*n =* 54)**	
**IRI-Interpersonal Reactivity Index, mean (SD)**					
- PT-Perspective-Taking Scale (7–35)	24.4 (5.3)	24.2 (4.3)	23.9 (2.7)	24.6 (3.44)	ANOVA: *F =* 0.202; *p =* 0.895
- FS-Fantasy Scale (7–35)	20.4 (4.3)	21.4 (4.2)	20.7 (4.2)	21.3 (4.2)	ANOVA: *F =* 0.640; *p =* 0.590
- EC-Empathic Concern Scale (7–35)	**23.3 (6.1)^§^**	26.2 (5.02)^**^	24 (3.8)	26.8 (3.9)^**^	ANOVA: *F =* 5.836; *p =* 0.001
- PD-Personal Distress Scale (7–35)	**15.3 (4.5)^@^**	18.0 (4.8)^**^	**15.4 (4.1)^@^**	18.5 (4.2)^**^	ANOVA: *F =* 10.804; *p =* 0.000
**PHQ – Patient Health Questionnaire total score, mean (SD)**	5.2 (3.9)	5.9 (4.1)	6.1 (4.43)	7.5 (4.8)	ANOVA: *F =* 2.467; *p =* 0.063
**HIS – Hope Integrative Scale total score, mean (SD)**	84.7 (13.1)	87.1 (11.7)	87.6 (12.1)	84.1 (11.4)	ANOVA: *F =* 1.058; *p =* 0.368
**HIS – Hope Integrative Scale dimension score, mean (SD)**					
- Trust and Confidence (9–54)	31.1 (5.8)	32.1 (4.9)	32.2 (5.6)	31.2 (5.6)	ANOVA: *F =* 0.546; *p =* 0.651
- Social Relationship and Personal Value (4–24)	14.1 (3.5)	15.1 (2.3)	14.8 (2.1)	14.4 (2.5)	ANOVA: *F =* 1.950; *p =* 0.123
- Positive Future Orientation (4–24)	17.5 (2.5)	18.1 (1.9)	18.2 (1.8)	18.0 (1.8)	ANOVA: *F =* 1.030; *p =* 0.380
- Lack of Perspective (6–36)	8.1 (4.5)	8.2 (5.2)	7.7 (5.3)	9.5 (4.8)	ANOVA: *F =* 0.202; *p =* 0.895
**UCLA Loneliness Scale total score, mean (SD)**	10.6 (4.6)^**^	13.2 (4.6)	10.9 (5.6)^**^	**15.3 (5.1)^#^**	ANOVA: *F =* 9.401; *p =* 0.000

** *p = < 0.001*.

§ *post-hoc Bonferroni test: MS and HPS women differ from MS men (highlighted in bold text)*.

@ *post-hoc Bonferroni test: MS and HPS women differ from MS and HPS men (highlighted in bold text)*.

# *post-hoc Bonferroni test: MS and HPS men differ from HPS women (highlighted in bold text)*.

### Patient Health Questionnaire (PHQ-9)

The HPS group had higher scores on the PHQ-9 than the MS group, with a statistically significant larger proportion of more than 20% of the HPS group suffering from depressive symptoms ranging from mild to severe levels compared to ~10% of the MS group. No statistically significant difference was found based on gender (Tables 3, 4).

### Integrative Hope Scale (IHS)

The sense of hope, identified across the four domains of the IHS scale (trust and confidence, social relationships and personal value, positive future orientation, and lack of perspective), did not significantly differ between the two MS and HPS groups or across genders.

### UCLA Loneliness Scale

Regarding the feeling of loneliness, the HPS group showed significantly higher scores than the medical student group (Table 3). Regarding gender differences, the female students belonging to the HPS group complained of a more intense feeling of loneliness than the male MS (*post-hoc* Bonferroni test, mean difference = 4.683; *p* < 0.000) and male HPS (mean difference = 4.402; *p* < 0.001) groups (Table 4).

### Predictors of Empathy

The logistic regression analysis results for identifying predictors of the cognitive dimensions of empathy are reported in Table 5.

**Table 5:** Logistic regression analysis for predicting the cognitive dimensions of empathy as measured by Interpersonal Reactivity Index (IRI).

	**IRI - Perspective Taking Scale higher scores (75th percentile)**					**IRI - Fantasy Scale higher scores (75th percentile)**				
	**B**	***p***	**Exp(B)**	**CI 95% L**	**CI 95% U**	**B**	***p***	**Exp(B)**	**CI 95% L**	**CI 95% U**
**Sex**	−0.39	0.295	0.677	0.326	1,405	−0.022	0.951	0.978	0.49	1,953
Men/Women										
**Age** ***<*** **21 years**	1,529	0.012	**4,612**	1,395	15,242	0.425	0.414	1.53	0.552	4,243
No/Yes										
**Large family (5 or more members)**	−0.307	0.475	0.735	0.317	1,708	0.186	0.622	1,204	0.576	2,519
No/Yes										
**Father' university degree**	−0.036	0.932	0.965	0.425	2.19	−0.701	0.078	0.496	0.228	1,082
No/Yes										
**Mother' university degree**	−0.417	0.335	0.659	0.282	1,539	0.266	0.506	1,305	0.596	2,858
No/Yes										
**Low financial condition**	0.251	0.644	1,285	0.443	3,723	0.226	0.656	1,253	0.464	3,388
No/Yes										
**Health Professions student**	1,155	0.025	**3,175**	1,154	8,734	0.309	0.481	1,362	0.577	3,217
No/Yes										
**Working student**	−0.751	0.188	0.472	0.154	1,444	0.247	0.601	1.28	0.508	3,228
No/Yes										
**Previous contacts for psychological problems**	0.861	0.068	2,366	0.939	5.96	0.346	0.323	1,414	0.711	2,813
No/Yes										
**PHQ-9 score** ***>*****10**	−0.4	0.504	0.67	0.207	2,167	0.778	0.072	2,178	0.934	5,081
No/Yes										
**UCLA Loneliness Scale total score**	−0.028	0.48	0.973	0.901	1.05	0.185	0.708	1,203	0.457	3,169
**IHS - social relationships and personal value**	0.102	0.17	1,107	0.957	1,281	0.003	0.941	1,003	0.933	1,077
No/Yes										

*PHQ-9, Patient Health Questionnaire; HIS, Integrative Hope Scale. Statistically significant predictors (highlighted in bold text)*.

Being an HPS increased the likelihood of showing a higher empathetic ability on the cognitive dimension of “perspective taking” by more than 3 times (OR, 3.175), and being 21 years old or under seemed to increase the likelihood of expressing competence by more than 4 times (OR, 4.612) on the same dimension. None of the variables entered in our model were statistically significant predictors of the high expression of cognitive empathy, as defined by the “fantasy scale” of the IRI.

Table 6 illustrates the results of the predictors of the affective dimensions of empathy. In our sample, the feeling of hope related to social relationships and personal value increased the likelihood of showing high “emphatic concern” affective abilities almost 1 and a half times. The strongest predictor was gender, with the female students having more than 3 times (OR, 3.111) the likelihood of showing a more compassionate attitude than the male students. Higher mothers' level of education (OR, 2.764) and higher students' depressive symptomatology (OR, 2.777) increased the likelihood of the student becoming more skilled in “emphatic concern” affective abilities for both variables by almost 3 times.

**Table 6:** Logistic regression analysis for predicting the affective dimensions of empathy as measured by Interpersonal Reactivity Index (IRI).

	**IRI - Emphatic Concern Scale higher scores (75th percentile)**					**IRI - Personal Distress Scale higher scores (75th percentile)**				
	**B**	***p***	**Exp(B)**	**CI 95% L**	**CI 95% U**	**B**	***p***	**Exp(B)**	**CI 95% L**	**CI 95% U**
**Sex**	1,135	0.009	**3,112**	1,328	7,288	0.38	0.285	1,463	0.729	2,936
Men/Women										
**Age** ***<*** **21 years**	0.093	0.873	1,097	0.354	3,403	0.315	0.563	1.37	0.472	3,976
No/Yes										
**Large family (5 or more members)**	−0.098	0.815	0.906	0.398	2,065	0.328	0.404	1,389	0.643	3
No/Yes										
**Father' university degree**	−0.69	0.122	0.501	0.209	1,204	−0.512	0.186	0.599	0.28	1.28
No/Yes										
**Mother' university degree**	1,017	0.023	**2,764**	1,147	6,659	0.446	0.27	1,562	0.707	3,451
No/Yes										
**Low financial condition**	0.297	0.605	1,346	0.436	4,156	−0.265	0.617	0.767	0.271	2,167
No/Yes										
**Health Professions student**	0.032	0.948	1,033	0.387	2,756	0.066	0.884	1,068	0.441	2,587
No/Yes										
**Working student**	0.095	0.855	1,099	0.399	3,028	−0.591	0.258	0.554	0.199	1,544
No/Yes										
**Previous contacts for psychological problems**	0.386	0.435	1,471	0.558	3,876	1,183	0.017	**3,263**	1,238	8,601
No/Yes										
**PHQ-9 score** ***>*****10**	1,021	0.049	**2,777**	1,004	7,681	0.55	0.285	1,733	0.633	4,744
No/Yes										
**UCLA Loneliness Scale total score**	0.042	0.304	1,043	0.962	1.13	0.165	0.000	**1.18**	1.09	1,276
**IHS - social relationships and personal value**	0.313	0.000	**1,367**	1,152	1,622	0.102	0.13	1,108	0.97	1,265

*PHQ-9, Patient Health Questionnaire; HIS, Integrative Hope Scale. Statistically significant predictors (highlighted in bold text)*.

Risk factors associated with lower emphatic skills, as identified by the higher scores on the IRI personal distress scale, were represented by previous contact for psychological problems (OR, 3.263) and the students' feelings of loneliness (OR, 1.18). Both of these variables, i.e., personal psychological distress and the perception of isolation, seemed to increase the risk of poor prosocial behavior.

## Discussion

The current study aimed, first, to investigate the sociodemographic, psychological, and psychosocial characteristics of MS and HPS and their level of empathy. Due to the growing emphasis on interprofessional collaboration within health care systems and the findings that empathy is associated with positive clinical outcomes (Hojat et al., 2011; Del Canale et al., 2012), it is important to consider and examine empathy levels across health disciplines.

Second, we wanted to test a psychosocial predictive model of their empathic skills.

To the best of our knowledge, this is the first study to investigate potential predictors of empathic abilities in these two populations of students along two cognitive and affective dimensions according to a model incorporating personal and environmental factors.

First, regarding the sociodemographic, psychological, and psychosocial findings related to MS and HPS, we found that the two populations, both of which were mostly represented by women, showed significant differences in their families' and contextual characteristics. The HPS lived in larger families, with a lower parents' education and with lower financial conditions than the MS. Compared to the MS, the HPS were younger (as foreseen, because of the different duration of their degree courses), and almost 20% of them were working students.

Despite the data in the literature reporting that MS suffer from depression to a greater extent than students attending other university courses (Singh et al., 2016), our findings showed that the HPS were more depressed and lived with more intense feelings of loneliness than the MS. Our study showed that ~10% of the MS suffered from depression, a proportion much lower than the 27.2% reported by Rotenstein et al. (2016) and the 39.1% reported by Atienza-Carbonell and Balanza-Martinez (2020). Given the heterogeneity among studies regarding the prevalence of depression between male and female medical students, our findings reported no difference, consistent with several previous studies (Puthran et al., 2016; Rotenstein et al., 2016; Pacheco et al., 2017).

In contrast to our hypothesis, compared to the MS, the HPS included in our study showed a higher proportion (23.3%) of students reporting depressive symptomatology and feelings of loneliness, as measured by the UCLA Loneliness Scale. When investigated by gender, this finding should be attributed only to female HPS. There is a growing number of studies documenting the decreased psychological well-being of students in professional programs (Ying, 2008). Although the percentage of our depressed HPS was higher than that of our MS, the prevalence of depression in our HPS was much lower than the alarming prevalence of more than 40% for applied medical science and nursing students shown by AlFaris et al. (2016). Our data are more consistent with those of Celik et al. (2019), who found that approximately one-fifth of the students showed depressive symptoms. Celik et al. reported that nursing and midwifery students with poor academic performance, poor economic status, smoking or alcohol use, chronic illness or mental problems were more likely to experience depression (Celik et al., 2019).

Confirming our hypothesis regarding empathetic and interpersonal abilities, no statistically significant difference was found between our two groups of students in cognitive and affective dimensions of empathy, but gender-based differences were identified. The women in both groups showed high levels of affective empathic skills, specifically in responses oriented toward another person in physical or emotional pain, a construct identified by the emotional aspect of empathy, “empathic concern” (Tone and Tully, 2014). Our findings showed how this response could also translate into a maladaptive form, especially for female MS (Park et al., 2015). Indeed, the women in both groups seemed to be particularly prone to personal distress empathic reactions, confirming previous findings of girls' sensitivity compared to boys' sensitivity related to better relationships with peers and, at the same time, greater vulnerability to experiencing distress in the face of others' discomfort (Shamay-Tsoory et al., 2009).

Similar to other studies (Hojat et al., 2002; Chen et al., 2007; Kataoka et al., 2009; Fields et al., 2011; Magalhaes et al., 2011; Petrucci et al., 2016; Quince, T. A et al., 2016; Williams et al., 2016), the results of our study highlighted that the female students had significantly higher average empathy scores than the male students.

Second, we tested a psychosocial predictive model of empathic skills in our sample of students. Regarding empathy measures, we focused and differentiated attention on the affective and cognitive dimensions to identify predictors of the presence development of each specific skill, given that these two abilities can follow a different developmental course.

In our sample, the logistic regression analysis results to identify predictors of cognitive empathic abilities did not identify any statistically significant predictor for the fantasy scale dimension. Instead, being 21 years old or under seemed to increase by more than 4 times the likelihood of showing a higher emphatic ability to take the mental perspective of others, which allows one to make inferences about their mental or emotional states (perspective taking dimension). Being an HPS increased the likelihood of showing competence in this dimension by more than 3 times. We cannot compare our results with several literature findings showing that cognitive empathy declines more with age than affective empathy. Our study was not longitudinal and did not assess empathic abilities in different cohorts of students (Gruhn et al., 2008; Sze et al., 2012). In the literature, these types of results have been inconsistent: although cognitive and affective empathy levels seem to be significantly higher in students aged 17–19 than in students aged 20–24 (Wang et al., 2019), other authors have reported higher levels of cognitive and affective empathy in middle-aged adults than in young people (O'Brien et al., 2013). In our study, the status of the HPS was associated with a relatively more disadvantaged family socioeconomic background, and we might speculate that the HPS were more prone to mentalize others' states of pain. Several scientific contributions have studied the impact of low socioeconomic status, associated with better accuracy at determining others' emotional states (Kraus et al., 2010) and with greater self-reported compassion for others, and shown a more pronounced heart rate deceleration in response to videos of others in compassion-inducing situations (Stellar et al., 2012). In addition, low socioeconomic status has been shown to be associated with more charitable and prosocial behavior (Piff et al., 2010, 2012; Davis et al., 2019). To strengthen these data, Varnum et al. (2015) showed that empathy was negatively related to a person's higher socioeconomic status with diminished neural empathic responses, although higher socioeconomic status was positively correlated with self-reported trait empathy, suggesting that those higher in status may not realize that they are actually lower in empathy (Varnum et al., 2015).

Not surprisingly, female gender was the strongest predictor of affective empathic abilities in our sample, with a >3 times higher likelihood of showing a compassionate attitude *(“I feel what you feel”)*. Our predictive model showed that higher levels of the mothers' education and students' depressive symptomatology increased the likelihood of a student becoming more skilled in the “emphatic concern” affective ability by almost 3 times. Similarly, Hasan et al. (2013) found a statistically significant association between empathy and the educational level of the mother. Our findings did not confirm that students whose mothers held college and above qualifications were less likely to suffer from burnout problems, which was in turn associated with a lower level of empathy than students whose mothers' educational level was primary school and below (Wang et al., 2019). We can suppose that Italian mothers' high education level could promote better emotional recognition and regulation based on the growing importance of socioaffective education from early childhood development. Additionally, in our sample, the feeling of hope related to social relationships and personal value increased almost 1 and a half times the likelihood of showing a higher ability to share the emotional experiences of others, i.e., a visceral reaction to their affective states (emphatic concern).

Finally, previous contact for psychological problems and feeling lonely would seem to represent risk factors for lower empathic skills, as identified by the higher scores on the IRI personal distress scale. Previous contact for psychological problems seemed to increase the likelihood of expressing maladaptive affective involvement by more than 3 times. Approximately 15% of both the MS and HPS reported previous contact for psychological problems. Given a preexisting psychological vulnerability, a stressful, demanding, and competitive context can contribute to the manifestation of a poor reaction to others' emotional states. Our findings are consistent with recent studies with healthcare professionals indicating an inverse association between empathy and loneliness (Marilaf Caro et al., 2017; Soler-Gonzalez et al., 2017; Berduzco-Torres et al., 2020), and in our sample, feeling alone increased the likelihood of showing a distressed empathic investment by almost 1.2 times.

Our results identify potential risk factors mediated by several other biopsychosocial variables not considered in this study. Low empathy levels could be considered negative in medical schools, since empathy may be the most powerful tool for a successful collaboration between the users and the professionals. The levels of empathy can show different trajectories and variations were found among medical students across the number of educational years, influencing specialty preferences (Andersen et al., 2020). Moreover, the likelihood of reporting lower empathic levels of MS and HPS due to the previous contact for psychological problems and feeling lonely could suggest a further explanation: the IRI self-assessment could have been influenced by biased cognitive processing of poor self-judgment related to depression (Dunn et al., 2009).

Some limitations of the present study should be acknowledged. First, the study was conducted in a single institution on a limited number of health professional courses. Second, all the measures were self-reported. The lack of correlation between self-assessed empathy levels and patients' perceptions or reality observed by others in the literature suggests that patients should be included in the process of empathy evaluation (Bernardo et al., 2018). Third, no comparison was performed across different years of the course of education that would be useful for understanding empathic changes over time; fourth, gender non-homogeneity of the sample was a limitation.

## Conclusions

Empathy is by nature multidimensional, interpersonal, and modulated by context (Decety, 2020). The influence of medical training conditions on empathy is still underresearched (Pedersen, 2009). International research and recommendations have repeatedly emphasized the importance of helping medical students develop and increase their empathy (Seitz et al., 2017).

Our exploratory study with Italian medical and health professional students provides a picture of different profiles between MS and HPS. Additionally, it investigated the “heart and head” empathy predictors of these two student populations during academic life (Ahrweiler et al., 2014; Quince, T. et al., 2016). Our findings emphasize the need to test psychosocial models to better understand empathic skills.

Medical and health professional course teachers could be encouraged to develop tools to increase student trainees' empathy levels. In our sample, modifiable factors, such as reducing depression and feelings of loneliness and increasing the sense of hope, could represent the goal of targeted psychological interventions that could impact and improve affective “heart” empathy.

This approach will have important implications for medical school training, as maintaining or increasing empathy levels is essential for producing physicians and health professionals with enhanced service user–health professional relationship styles.

## Data Availability Statement

The raw data supporting the conclusions of this article will be made available by the authors, without undue reservation.

## Ethics Statement

The studies involving human participants were reviewed and approved by University of L'Aquila, Internal Review Board, approval number 20/2020. The patients/participants provided their written informed consent to participate in this study.

## Author Contributions

LG, MC, and RR designed the study, wrote the protocol, conducted literature research, and wrote the publication. LG and RR conducted the statistical analyses. SM, AS, DU, and DB collected data and their systematization in a database. All authors contributed to the article and approved the submitted version.

## Conflict of Interest

The authors declare that the research was conducted in the absence of any commercial or financial relationships that could be construed as a potential conflict of interest.
